# The Shape of the Lymphocyte Receptor Repertoire: Lessons from the B Cell Receptor

**DOI:** 10.3389/fimmu.2013.00263

**Published:** 2013-09-02

**Authors:** Katherine J. L. Jackson, Marie J. Kidd, Yan Wang, Andrew M. Collins

**Affiliations:** ^1^School of Biotechnology and Biomolecular Sciences, University of New South Wales, Sydney, NSW, Australia

**Keywords:** BCR repertoire, TCR repertoire, V(D)J recombination, public clonotypes, private clonotypes, combinatorial diversity, junctional diversity

## Abstract

Both the B cell receptor (BCR) and the T cell receptor (TCR) repertoires are generated through essentially identical processes of V(D)J recombination, exonuclease trimming of germline genes, and the random addition of non-template encoded nucleotides. The naïve TCR repertoire is constrained by thymic selection, and TCR repertoire studies have therefore focused strongly on the diversity of MHC-binding complementarity determining region (CDR) CDR3. The process of somatic point mutations has given B cell studies a major focus on variable (IGHV, IGLV, and IGKV) genes. This in turn has influenced how both the naïve and memory BCR repertoires have been studied. Diversity (D) genes are also more easily identified in BCR VDJ rearrangements than in TCR VDJ rearrangements, and this has allowed the processes and elements that contribute to the incredible diversity of the immunoglobulin heavy chain CDR3 to be analyzed in detail. This diversity can be contrasted with that of the light chain where a small number of polypeptide sequences dominate the repertoire. Biases in the use of different germline genes, in gene processing, and in the addition of non-template encoded nucleotides appear to be intrinsic to the recombination process, imparting “shape” to the repertoire of rearranged genes as a result of differences spanning many orders of magnitude in the probabilities that different BCRs will be generated. This may function to increase the precursor frequency of naïve B cells with important specificities, and the likely emergence of such B cell lineages upon antigen exposure is discussed with reference to public and private T cell clonotypes.

## Germline Genes and Lymphocyte Diversity

The mammalian immune system has the ability to respond to almost any antigen to which it is exposed because of the incredible diversity of lymphocyte receptor molecules. The diversity of both the B cell receptor (BCR) repertoire and the T cell receptor (TCR) repertoire is made possible by multiple sets of highly similar genes that recombine to form functional genes. Immunoglobulin heavy chains are encoded by recombined VDJ genes that are formed from sets of Variable (V), Diversity (D), and Joining (J) genes (IGHV, IGHJ, IGHD), while VJ rearrangements of kappa and lambda chain V genes (IGKV, IGLV) and J genes (IGKJ, IGLJ) encode the immunoglobulin light chains (1, 2). TCR β-chains and δ-chains are similarly encoded by distinct sets of V, D, and J genes (TRBV, TRBD, TRBJ; TRDV, TRDD, TRDJ), while α-chains and γ-chains are encoded by additional sets of V and J genes (TRAV, TRAJ; TRGV, TRGJ) (3–5). The resulting combinatorial diversity is expanded still further by junctional diversification arising from exonuclease trimming of the recombining gene ends and from the essentially random addition of nucleotides, between the recombining genes, by the enzyme terminal deoxynucleotidyl transferase (TdT) (6). Together, combinatorial diversity and junctional diversity create the diversity of the naïve T cell and B cell repertoires. Limitations to diversity may however be a feature of V(D)J rearrangement that is as significant to immune function as the bewildering number of lymphocyte specificities that can theoretically be generated.

This review will present evidence that biases in the processes that generate combinatorial and junctional diversity are such that the probabilities of different BCRs and TCRs being generated is highly variable. This results in B and T cells of some specificities being present within the naïve repertoire at high frequency, while other specificities may or may not be present at all. The unevenness of the receptor abundance distribution can be said to give “shape” to the naïve B and T lymphocyte repertoires. This distribution may be further shaped by processes including positive and negative selection, clonal expansion and, in the case of immunoglobulin genes, by somatic hypermutation, however this review will focus upon recombination and gene processing.

**Figure 1:**
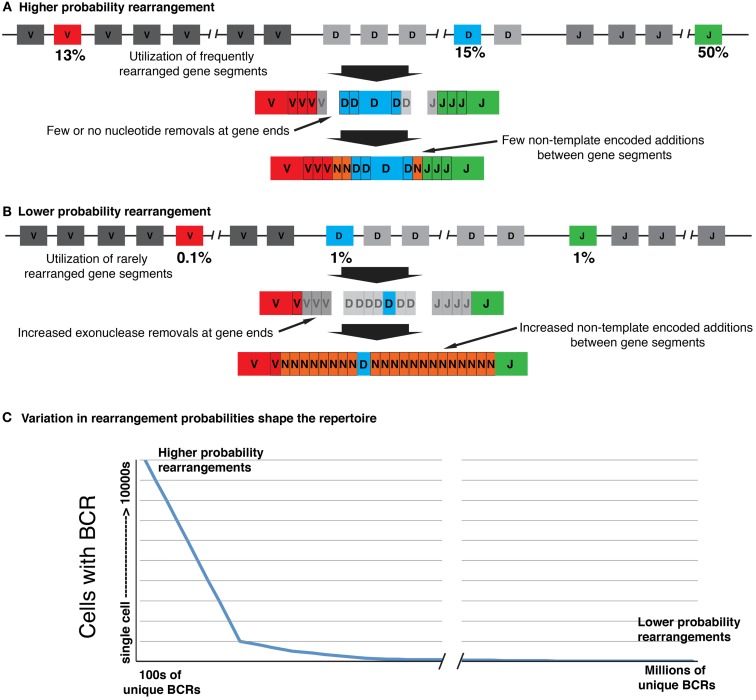
**The receptor repertoire has “shape,” as biases and constraints in the recombination process vary the probabilities of generating particular V(D)J rearrangements**. **(A)** Higher probability rearrangements are generated through utilization of more frequently rearranged gene segments. These segments are joined with minimal gene processing and N-addition, increasing the chance of independent rearrangements with identical or near identical CDR3s. **(B)** Lower probability rearrangements utilize rarely rearranged germline gene segments and the CDR3s are more diverse owing to increased nucleotide removals and additions at the joins. **(C)** The many order of magnitude differences in the likelihood of generating particular rearrangements shape the repertoire, with higher probability rearrangements being frequently generated and as a consequence being carried by many identical B-cells. Only a relatively small number of unique rearrangements will be generated with probabilities high enough to be carried by a large number of B cells, but this should ensure that they are always present in the repertoire at significant levels. Conversely, lower probability rearrangements may be so rare that they are carried only by a single B cell, or are entirely absent from the repertoire. The lower probability rearrangements that are carried by just one or at most a few B-cells likely represent many millions of unique rearrangements.

As the shape of the naïve human B and T cell lymphocyte repertoire is an outcome of the evolution of genetically determined biases, this should ensure the presence of critical rearrangements in the repertoire of all individuals. It should also ensure that these critical rearrangements are carried by multiple naïve cells (see Figure 1). Such populations of specific naïve lymphocytes will have a competitive advantage during antigen-driven clonal selection, and any discussion of repertoire diversity that is limited to the size of the population of unique receptors will therefore be ignoring a parameter of likely biological significance. In this review, we will use the term “repertoire” to refer to the complete set of receptors that are carried by an individual, including multiple copies of particular sequences. The number of unique sequences that are found within an individual’s repertoire will be described as the “diversity” of the repertoire.

The size of the sets of germline genes make a major contribution to lymphocyte diversity, but surprisingly, our knowledge of these germline genes is far from complete. In part this is the result of the complexity of the loci, for they feature numerous highly similar genes that are thought to have evolved via gene conversion (7), and duplication and divergence (8). These genes are interspersed with many pseudogenes and repetitive elements (8). Sequencing and annotation of the loci is therefore challenging. These complexities also mean that SNPs arising from short read-length sequences generated in studies such as the HapMap and 1000 Genomes projects, cannot be used for the imputation of full-length allelic variants. In fact, these projects utilize polyclonal lymphoblastoid cells lines in which the immunoglobulin loci have undergone somatic recombination, and the rearranged genes may have been affected by somatic point mutation. This makes these cell lines unsuited to the study of immunoglobulin genes (9).

Arguably, it is the BCR germline genes that are best known, and paradoxically, this is because of their transformation through the process of somatic hypermutation, during an immune response. IGHV genes are by far the longest of the recombining IGH genes, and they are the principal targets of the mutational machinery (10, 11). Many studies of the immunogenetics of immunoglobulin have therefore concentrated upon the IGHV genes. As it is necessary to be certain of the germline origin of mutated sequences, if accurate studies of point mutations are to be conducted, the complete and accurate definition of the set of germline immunoglobulin IGHV genes and allelic variants has been and should remain a focus of research.

The official human IGHV germline gene dataset, curated by the ImMunoGeneTics (IMGT) group, includes 129 functional genes, open reading frames (ORF), and pseudogenes, as well as over 200 allelic variants (12). Interest in these germline genes has increased in recent years, resulting in 40 new allelic variants being reported since 2005 (13–17). Many additional IGHV allelic variants have also been identified in recent high-throughput sequencing studies, through analysis of cDNA-derived VDJ gene rearrangements (18, 19), but these have not been accepted as part of the official IGHV dataset. We have designated alleles identified in this way with unofficial allele names using an indicator (“p”) of their “putative” nature (e.g., IGHV3-9*p03) (15), and these additional alleles can be found in the UNSWIg repertoire (http://www.ihmmune.unsw.edu.au/unswig.php).

The official human light chain V gene datasets appear to be relatively complete and accurate, though few allelic variants have been reported (20). Nevertheless these few variants appear to be of functional and clinical significance. For example, a variant kappa gene allele was identified within the Navajo population and has been reported to account for the susceptibility of this population to infections (21).

The human IGH germline genes receive continuing attention while the IMGT human TCR germline gene datasets have barely changed since the complete sequences of the TCR gene loci were first described (22, 23). The IMGT TRBV dataset includes 65 functional genes, ORFs and pseudogenes, and just 13 allelic variants, and no new TRBV sequence has been added to the dataset since the publication of the complete sequence of the TRB locus in 1996 (22). Only three TRAV/TRDV sequences (24) in the IMGT dataset are derived from studies published since the reporting of the complete sequence of the TRAV/TRDV locus (23), and some variants that were described soon afterward still remain officially unrecognized (25). The incomplete nature of the IMGT TRBV, and TRAV datasets in particular are clearly highlighted in the literature, for sequencing studies have reported many SNPs in the coding regions of these genes. Subramanyan and colleagues reported 279 SNPs in a study of 63 TRBV genes in 10 individuals from each of four human populations (26). Of these reported SNPs, 114 were located in coding regions of functional TRBV genes (26). A similar study of 57 TRAV/TRDV genes in the same 40 individuals resulted in the discovery of 284 SNPs, 51 of which encode amino acid changes in the coding regions of the gene sequences (27). The allelic variants associated with these TRAV/TRDV and TRBV SNPs have not been reported in the literature or in sequence databases, and they have not been incorporated into the official gene datasets. This is surprising because the SNPs were identified through amplification and sequencing of full-length genomic sequences. It is also unfortunate, for studies of TCR polymorphisms have shown that they can be of functional significance (28, 29).

The BCR and TCR D loci contribute differently to the generation of diversity, and the differences in the nature of the loci have influenced BCR and TCR research directions. The 27 human IGHD genes include 25 functional genes, 23 of which are unique (30). Although some IGHD genes, especially those of the IGHD1 gene family, are very similar, there is considerable sequence diversity amongst the genes. The lengths of the IGHD genes vary from 11 nucleotides to 37 nucleotides, and almost all of them are substantially longer than the TRBD and TRDD genes. This length and the IGHD gene variability have made improvement in the identification of IGHD genes within VDJ rearrangements a challenging but achievable research goal. Pursuit of this goal has driven the development of immunoglobulin gene alignment utilities including SODA2 (31), IgBLAST (32), and iHMMune-align (33). The objective measurement of the performance of these utilities is made difficult, however, by a lack of appropriate data sets. Ideally, performance would be measured using rearranged sequences of known composition. As such sets are unavailable, clonally related sequences can be used (32, 33). We have also compared the performance of different utilities using a set of long-read pyrosequenced (Roche 454) IGH rearrangements from an individual with a homozygous deletion of six IGHD genes (34). This test measures performance by the number of VDJ rearrangements in the dataset that are said to include the absent IGHD genes. Together these studies demonstrate that IGHD genes can now be identified with confidence, and as a consequence, analysis of the BCR heavy chain complementarity determining region (CDR) 3 can include detailed analysis of IGHD gene usage, gene processing, and N nucleotide addition.

Analysis of the TCR CDR3 is not so easy. The two human TRBD genes are both short (12 and 16 nucleotides) and highly similar at their 5′ ends (22). This makes their identification in VDJ rearrangements particularly difficult. The TRBD genes within a VDJ rearrangement are likely to be flanked by N-REGIONS of non-template encoded nucleotides. These nucleotides are introduced through the action of the TdT enzyme, which is biased to the addition of guanine (G) nucleotides (35) and to the addition of homopolymer tracts (36, 37). Distinguishing TRBD gene ends from G-rich N nucleotides is difficult because the TRBD genes are G-rich at both their 5′ and 3′ ends. A final complication is that the two alleles of TRBD2 differ by just a single nucleotide. This critical nucleotide is flanked on both sides, in both alleles, by GGG motifs. For these reasons, few TCR studies have included detailed analysis of TRBD genes and their processing, or of the N-REGIONS that can only be defined after the identification of a TRBD gene segment within the CDR3. Even the most recently developed TCR alignment utility excludes identification of TRBD genes from its output (38).

Analysis of the VDJ junction in TRD rearrangements is equally difficult. The three human TRDD genes are just 8, 9, and 13 nucleotides in length (4). This makes their reliable identification within VDJ rearrangements especially problematic if nucleotides have been lost through exonuclease activity. Application of an approach previously used in the analysis of BCR sequences (37) suggests that eight nucleotides is the minimum D gene length that will allow TRDD genes to be reliably distinguished from N-REGIONS within a junction of 12 or fewer nucleotides, while 9 nucleotides are needed for regions from 13 to 15 nucleotides and 10 nucleotides for junctions greater than 15 nucleotides (Jackson, unpublished data). It is therefore no surprise that few studies have reported the partitioning of TRD junctions as two of the three TRDD genes can only be confidently delineated from N-additions in their unprocessed form.

The J loci of the human BCR and TCR also include important differences. The IGHJ locus includes six functional genes, which are all found downstream of the IGHD locus in a single cluster. Allelic variants have been reported for IGHJ3, IGHJ4, IGHJ5, and IGHJ6, though there is reason to doubt the existence of the reported allelic variants of IGHJ3 and IGHJ5 (39). TCR J genes are more numerous and are differently organized. The TRBJ genes are found as a block of six genes located downstream from the TRDB1 gene, and a block of seven genes located downstream from the TRDB2 gene. The TRDB1 gene can pair with all J genes, but the TRDB2 gene is strongly biased toward pairing with its associated J genes (40). There are also four functional J genes in the TRDJ locus. Functional allelic variants have only been reported for the TRBJ1-6 gene.

## Biases in Combinatorial Diversity and the Shaping of the Repertoire

Combinatorial diversity is that part of repertoire diversity that results from the fact that functional receptor genes form by the recombination of members of the sets of germline V, D, and J genes. This diversity is usually calculated by simply multiplying together the number of functional V, D, and J genes that are available within the genome. Such calculations, however, may promote misunderstandings, for they encourage the view that “all genes are equal,” and that all combinations are equally likely. TCR studies have paid considerable attention to capturing an unbiased sampling of the repertoire, for example using 5′ RACE to amplify TCR transcripts from the constant region gene. Such studies have shown that TCR genes are highly biased in their usage (41–43). In contrast, many BCR repertoire studies have amplified both mRNA and genomic rearrangements, often using IGHV gene family-targeting primer sets that were developed for the detection of malignancies rather than for the investigation of the repertoire (44, 45). Such primers almost certainly lead to some distortions in the relative abundances of different sequences that are seen. Nevertheless, BCR studies utilizing different primer sets, and amplifying different source material are surprisingly consistent, and the B cell literature provides unequivocal evidence of strong gene utilization biases.

Different IGHV genes are used at frequencies that range from as little as 0.1% to more than 10% of all rearrangements in an individual’s naïve B cell repertoire (18, 46). Utilization frequencies also vary between alleles. For example, analysis of VDJ recombination in different individuals has shown that IGHV1-2*02 is used approximately three times as often as IGHV1-2*04, in individuals who carry both these alleles (18). IGHV utilization frequencies are surprisingly constant between individuals (47). Examples of such consistency include IGHV1-46 which varies from 2 to 3.1% in different individuals (average 2.65%), IGHV3-21 which varies from 3.5 to 6.3% (average 4.59%), and IGHV3-49 which varies from 0.8 to 1.3% (average 1.0%) (18). This is not true for all genes, with different individuals utilizing IGHV1-69 at frequencies that range from 3.1 to 9.1% (average 6.2%) (18). IGHV3-23, which is typically the most utilized IGHV gene, was seen on average in 6.7% of all VDJ sequences, but its utilization frequency in one individual was 13.7% (18).

Biased gene usage is not confined to the IGHV genes. IGHD gene usage varies from less than 1% (IGHD4-4/11) to over 15% (IGHD3-22) of total rearrangements. Biases in the resulting amino acid sequences of the CDR3 junction are even greater. IGHD segments can be utilized in all three reading frames, and each IGHD gene is therefore able to encode three distinct amino acid sequences. Analysis of IGH rearrangements in which the IGHJ is out-of-frame, and which are therefore non-productive, shows each IGHD gene rearranges at equal frequency in each of the three RFs, however among productive rearrangements there is a strong skewing of the utilization of each gene toward a dominant RF (48). This dominance is constant between individuals, and the preferred RF is gene family dependent. Analysis of in-frame and out-of-frame IGH rearrangements sequenced using the Illumina platform suggests that the underlying rearrangement processes have no reading frame bias, but that bias emerges from stronger negative selection of sequences in certain reading frames (48). Such negative selection particularly focuses on non-productive sequences that result from the presence of stop codons within the junction region. These are seen when many IGHD genes are translated in the non-dominant reading frame, and such genes can only be utilized in those reading frames if the stop codons are removed by exonuclease trimming. When analysis of IGHD usage in the expressed repertoire factors in the three RFs, the IGHD gene utilization frequencies span three orders of magnitude. There is also considerable variation between the utilization frequencies of IGHJ genes. The IGHJ4 gene is present in approximately 45–50% of rearrangements, while IGHJ6 accounts for a further 20–25% of VDJ rearrangements (49, 50). IGHJ1, on the other hand, is utilized by only 1% of all rearrangements (39).

Biases in light chain gene usage are just as strong. For IGK rearrangements, preferential inclusion of IGKV3-20 was noted in early studies of the expressed IGK repertoire of both adults and neonates (51–53), while single cell PCR (54) and bioinformatics analysis of IGK rearrangements from sequence databases showed IGKV3-15, IGKV3-11, IGKV1-5, IGKV2-30, and IGKV1-30/IGKV1D-39 to also display preferential rearrangement (20). These biases were confirmed again recently in a high-throughput sequencing study which also highlighted similarities in usage between individuals, including similarities between individuals from geographically distant and ethnically distinct populations (55). Under-utilization and over-utilization of the J gene segments have been reported. IGKJ1 and IGKJ2 appear more frequently, while there is under-utilization of IGKJ3 and IGKJ5 (20, 53, 54). This skewing of IGKJ usage toward the genes located 5′ in the IGKJ locus is seen despite the necessity for selection of more 3′ IGKJ genes during secondary IGK rearrangements (56, 57). A similar bias toward 5′ IGKJ genes is also seen in the mouse, and modeling of mouse light chain rearrangement supports the strong underlying tendency toward the initial rearrangement of IGKJ1 or IGKJ2 (58). The IGLV usage is strongly skewed toward a limited number of the functional V segments with 3 of the 30 IGLVs accounting for more than 50% of expressed rearrangements, and with individual IGLV segment frequencies ranging from 0.02 to 27% (59). Only four of the seven IGLJ are considered functional (60). The four IGLJ range from almost 55% utilization in the expressed B cell repertoire for IGLJ7, to just 5.5% for IGLJ1 (61).

Although bias in the reading frame of the IGHD gene is the result of selection, other biases appear to be intrinsic to the recombination process, for when analysis is confined to non-productive rearrangements which carry an out-of-frame J-REGION, preferential gene usage is still seen (48). Such sequences are not subject to positive or negative selection. The same biases have been observed among transcripts generated from transgenic mice that carry a human heavy chain mini-locus (62), while in NOD-scid-IL2Rγ null mice that had been reconstituted with human hematopoietic stem cells, typical patterns of biased usage were seen amongst the expressed light chain genes (63). Recent studies in monozygotic twins show that they share utilization frequencies for both the heavy and light chain genes (46, 63), with correlations in a similar range to replicate biological samples. When one twin was investigated following lymphocyte ablation therapy, the reconstituted repertoire showed the same utilization patterns (46). Unrelated individuals did not share this degree of correlation. The biases in utilization frequencies of different V, D, and J genes therefore appear to be genetically determined, and when acted upon by the recombination machinery, the biases in that process give rise to an individual’s distinct repertoire. Repertoire shape is therefore directly linked to the genotype of an individual’s immunoglobulin gene loci. This has become even clearer since high-throughput sequencing has allowed analysis to focus upon individual chromosomes.

**Figure 2:**
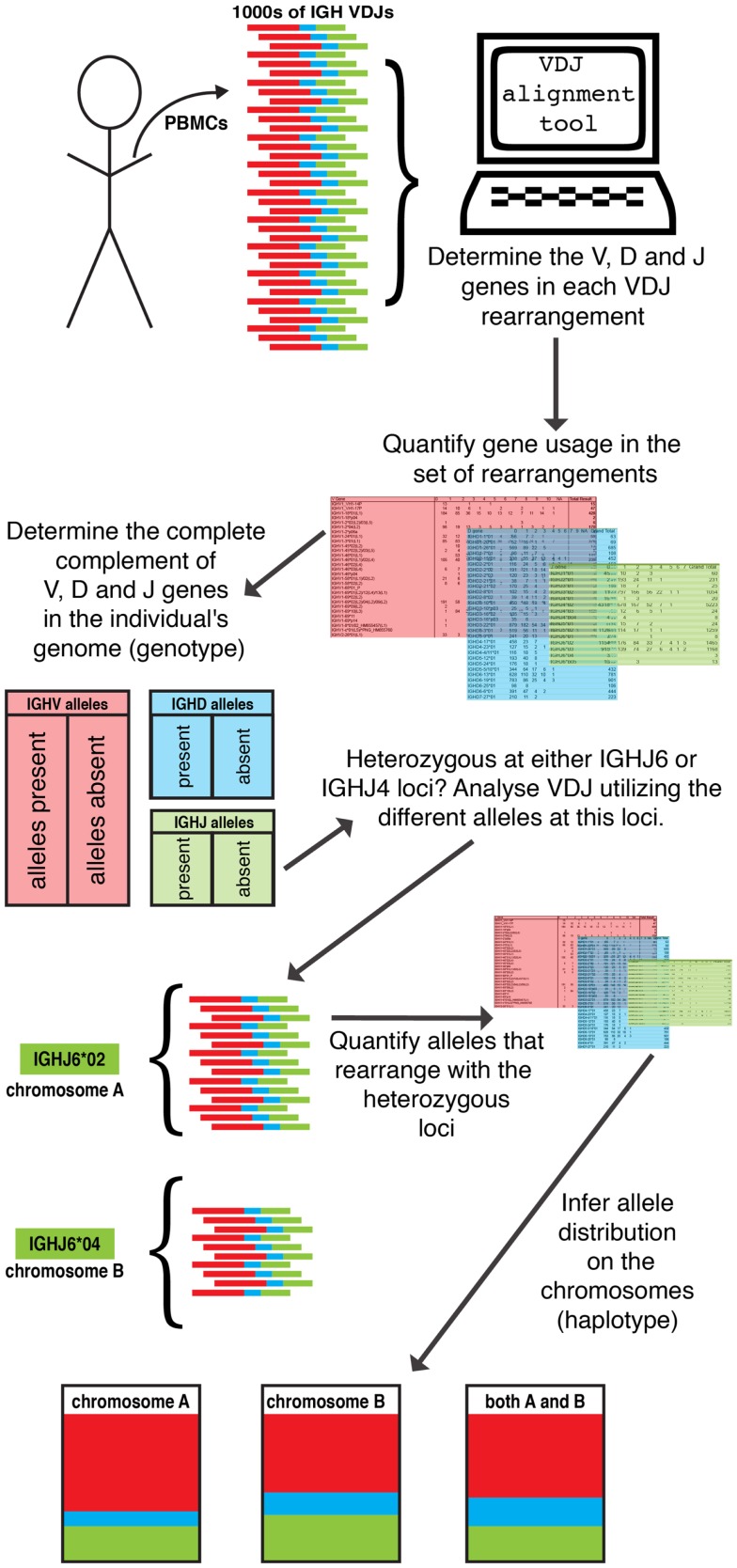
**Inference of IGH haplotypes from VDJ rearrangements**. The availability of large datasets of VDJ rearrangements from single individuals permit the inference of all germline V, D, and J genes within the genome, from analysis of apparent gene utilization within a dataset. As IGH VDJ rearrangement is an intra-chromosomal event, gene pairing in VDJ rearrangements can be used to infer which gene segments are carried by the same chromosome. Leveraging the heterozygous IGHJ6 or IGHJ4 locus allows the reconstruction of IGH gene segments on each chromosome and for the IGH haplotype to be inferred.

The large datasets that are now being generated by high-throughput sequencing from single individuals are facilitating analysis of the processes that shape the repertoire, but each dataset still represents a mixture of rearrangements from two independently recombining chromosomes. The fact that V(D)J rearrangement is an intra-chromosomal event, however, means that every V(D)J gene rearrangement provides information about the association of different genes on a chromosome. Any heterozygous locus allows each chromosome to be associated with one or the other allele at that gene locus, and large sets of V(D)J rearrangements can be analyzed to determine all the V, D, and J genes that rearrange on each chromosome. This allows the determination of inferred haplotypes (see Figure 2).

In practice, the complete inference of V, D, and J gene haplotypes by the analysis of V(D)J rearrangements is only likely to be possible in the case of the IGH locus. Approximately 40% of individuals are heterozygous at the IGHJ6 locus, and the IGHJ6 gene is present in nearly 25% of all rearrangements. It therefore provides an ideal “anchor-point” from which to haplotype the IGH locus. Using this approach, we recently investigated the IGH locus in nine individuals, and showed that all 18 IGH variable region gene haplotypes were unique (19). In addition to allelic variants, many IGHV and IGHD gene deletions and IGHV gene duplications were evident. The definition of haplotypes in this way is allowing IGH gene usage frequencies to be studied with unprecedented accuracy, but unfortunately no locus as appropriate as the IGHJ6 anchor-point exists amongst the light chain genes or amongst TCR genes. Limited investigations in the past have highlighted TCR haplotypic variation in the human population (64, 65), but the extent of variation within the IGH locus suggests that considerably more TCR variation may await discovery.

Many factors have been explored to explain biases in chromosomal recombination patterns. Variations in enhancers (66) have been implicated in biased murine TCR gene usage. Variations in recombination signal sequences (RSS) also influence utilization frequencies of both human BCR (67, 68) and TCR (69) genes. The IGKV polymorphism that has been linked to increased susceptibility to *Haemophilus influenzae* in the Navajo population includes a single nucleotide change in the heptamer sequence of the RSS, and it reduces recombination by 4.5-fold relative to the common allelic variant (21). The non-amer and heptamer sequences of the RSS are separated by either a 12 or 23 base pair spacer. Spacers also show sequence variation, and there has been debate about the impact this has on recombination efficiency. While some studies did not observe any impact when the regular spacer sequence was replaced with runs of GC pairs (70), competition assays using extra chromosomal substrates suggest differences in spacer sequence can result in differences in recombination efficiency that mirror differential gene usage in the V(D)J repertoire (67, 68). However, RSS variation cannot explain all differences in allele utilization. The recent re-sequencing of the complete IGH locus found that the IGHV-associated RSS were the same as those earlier reported by Matsuda (71) even where different alleles of the gene were present (17).

Some variation in the frequency with which particular gene sequences are seen in the repertoire may be explained by copy-number variations (CNV). The presence of CNV within the IG variable gene locus was first determined using sequence-specific RFLP analysis to determine gene copy-number (72), and the affect of CNV on expression levels was investigated through the examination of the binding of an anti-idiotypic monoclonal antibody (G6) to tonsillar IgD + B-cells (73). An examination of 35 individuals found that they carried between 0 and 4 copies of the IGHV1-69 gene. Linear regression determined that for each allele copy, approximately 3% of B-cells were G6 reactive. Individual differences in the IGHV1-69 copy-number could therefore result in the contribution that this single gene makes varying from being totally absent (0 copies) to being present in as many as 12% of rearrangements in individuals with four available copies.

Sequencing of single chromosomes of an individual’s IGH locus has now demonstrated that insertions, deletions, and complex events have altered the copy-number of IGHV genes, including the IGHV1-69 and IGHV3-23 genes (17). The duplicate IGHV3-23 genes remain within the genome as absolutely identical sequences. The presence of these and other CNVs has also been highlighted in bioinformatic studies of immunoglobulin genotypes (18) and haplotypes (19), where sequence data from single individuals clearly demonstrated that some individuals had more than two “alleles” of a single IGHV gene. Genes were also found to be absent from the genome of some individuals. A limitation of these bioinformatics studies was that gene duplications could only be detected if two distinct “allelic variants” were carried on a single chromosome.

In addition to the underlying biases in utilization of germline genes, a final bias has been identified that affects the contribution of recombination frequencies to repertoire diversity. For reasons that are presently unclear, there appear to be pairing preferences for some IGHD and IGHJ genes that increase the frequency of particular IGHD-IGHJ pairs within the repertoire. Biases were first observed in a small set of 59 non-productive rearrangements (74). Later analysis of 6,500 IGH VDJ sequences collected from public databases led to the observation that 5′ IGHD genes paired with increased frequency to the most 3′ IGHJ (J5/J6) and with decreased frequency to the 5′ IGHJ (J1–J4) (50). In contrast, 3′ IGHD tended to preferentially pair with 5′ IGHJ rather than 3′ IGHJ (50). This observation is also supported by analysis of very large datasets generated by pyrosequencing of VDJ rearrangements from three healthy subjects (75). Significantly more pairings were seen of IGHD2-2 and IGHD3-3 with IGHJ6, and of IGHD3-22 and IGHJ3 than would be predicted from the frequencies of these genes in the overall dataset (75).

The bias in D-J pairing also extends to the TCRB loci where the application of HTS approaches to murine TCRB repertoires has revealed a pattern of TRBD to TRBJ pairing that correlates to the genomic distance between rearranged genes (40). The TRBV and TRBJ gene usage in the mice was biased toward particular genes, but the pairings of TRBV and TRBJ were independent. The physical chromatin structure of the TRBD and TRBJ loci was investigated using a biophysical model of the chromatin conformation. The biases in TRBD to TRBJ pairing appeared to be better explained by this mechanical model than previously proposed genetic models based on RSSs (40). The model was also extended to human TRBJ usage with favorable evidence that chromatin conformation determines TRJB gene usage.

Biases in the pairing of heavy and light chains have also been reported. The existence of forbidden or unfavorable pairings of germline heavy and light chain genes was described in the early literature (76). This was not supported by later studies (77, 78), nor was it supported by a recent study that applied high-throughput sequencing to generate thousands of linked heavy and light chain genes (79).

## Biases in Junctional Diversity and the Shaping of the Repertoire

Both the naive B cell and T cell repertoires are limited in the periphery by processes of selection. However T cell selection within the thymus is a particularly rigorous process, and it leads to dramatic differences between the potential and the observed repertoire diversity. The idiosyncratic nature of TCR selection in a human population with abundant MHC diversity also means that analysis of the processes that contribute to TCR diversity will be difficult using datasets comprising sequences from multiple individuals. Sufficiently large datasets from single individuals with a specific MHC profile finally became available with the application of high-throughput sequencing to repertoire studies. However, the continuing difficulties involved with the identification of TCR D genes and hence the other constituent elements within the TCR CDR3 still discourage analysis of the genetic elements and processes that contribute to this region. It is therefore studies of BCR genes that provide the clearest insights into the processes that contribute palindromic P nucleotides and non-template encoded N nucleotides to the V(D)J junction, and into the process of exonuclease trimming that depletes the ends of recombining genes. A recent study of BCR CDR3 suggested that as a result of these processes, the circulating B cell population in a typical adult human includes 3–9 × 10^6^ unique heavy chain CDR3 (80).

Palindromic or P nucleotides are formed by the asymmetric opening of hairpin loops that form at gene ends during the rearrangement process (81). In the absence of exonuclease activity, the opening of the hairpins can add short, self-complementary single stranded extensions into the junctions. P nucleotide addition was first recognized as a process that can contribute to TCR CDR3 (82, 83), however the contributions of P nucleotides to the BCR repertoire have been more precisely quantified (84, 85). Similarly, it is recognized that N nucleotides make a major contribution to the diversity of both the TCR and BCR repertoire (86), but only BCR N-REGIONS have been subjected to detailed analysis (37). Where BCR studies have investigated the kinds of amino acids that are likely within N-REGIONS, studies of TCR N-REGIONS have focused upon analysis of the overall contribution of N-REGIONS to αβ TCR diversity. This has been studied in a comparison of wild-type mice with mice carrying homozygous null alleles for TdT (86). N-addition was estimated to contribute to 90% of the diversity of the αβ TCR repertoire (86). Diversity could be estimated in this and other studies because of the development of “spectratyping” techniques, which is the analysis of the CDR3 length distribution in PCR amplicons. It permitted some of the first explorations of the T cell repertoire, however it only allowed detailed analysis of N-REGIONS if further sequencing was undertaken. Until the advent of high-throughput sequencing, such analysis was usually compromised by the restricted number of sequences that could be generated from any individual, and by the challenges associated with D gene identification.

Non-template encoded N-additions are intrinsically biased owing to the preference of TdT toward the incorporation of G nucleotides. This is manifested in G/C-rich additions when viewing the N-REGIONS of the coding strand, as additions may be made to both the coding and non-coding strands during recombination. This has been demonstrated through analysis of extrachromosomal substrates transfected into human cell lines (36), as well as by analysis of human BCR (37) and TCR (87) VDJ rearrangements. The G/C bias is coupled with an apparent interdependence of the additions, which leads to the formation of homopolymer tracts (36, 37, 87). Together these biases ensure that the germline gene-encoded regions of the CDR3 are frequently flanked by amino acids such as glycine, that are encoded by G-rich codons (88). It has been proposed that the inclusion of small amino acids such as glycine, which has only a single side chain, promotes flexibility of the CDR3 loop (88).

Exonuclease trimming is perhaps the least understood process that contributes to the BCR and TCR repertoires. The mechanisms responsible for the loss of nucleotides from the coding ends of the genes during rearrangement remains to be determined, but a number of features of the process have been described, and intrinsic biases have been identified. The extent of processing from each gene end involved in a join (VD or DJ) is independent (87). That is, we do not see more processing on one side of the join to compensate for reduced processing of the gene on the other side. The processing differs for V, D, and J genes and for gene families. Removals may therefore be impacted by the sequence of the gene ends. Sequences with high A/T content appear more susceptible to nucleotide loss, while sequences with high G/C content appear resistant to processing (36, 84, 89, 90). This bias is still seen after controlling for the G/C bias of N-REGIONS.

**Figure 3:**
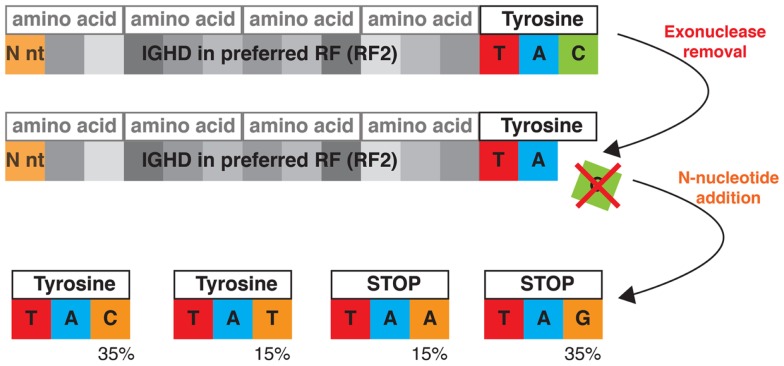
**Germline gene segment sequences constrain junctional diversity**. This is illustrated by processing of the codon TAC, which encodes tyrosine as the most 3′ amino acid in the preferred reading frame of many IGHD genes. A highly probable outcome of the VDJ rearrangement process is the loss of a single nucleotide from the IGHD coding end. If this occurs, the productive repertoire for these Ds is strongly skewed toward tyrosine, as two of the four possible nucleotide replacement events generate stop codons, and the other two ensure the maintenance of tyrosine. These nucleotides may be sourced from either N-addition or from the 5′ end of an IGHJ segment. The outcome of N-addition is depicted in the bottom panel. N-additions are colored in orange and below each of the four possible outcomes is shown the likelihood of the enzyme TdT adding each nucleotide base.

The gene sequence ends that remain after exonuclease processing provide a final bias that shapes the repertoire. The gene ends are constrained by the genetic code, to favor the formation of codons for a surprisingly limited number of amino acids. This is best illustrated in the case of the many IGHD genes that have the nucleotide sequence TAC at their 3′ end (see Figure 3). In the dominant reading frame, these nucleotides encode tyrosine. Removal of a single nucleotide creates a situation where only provision of a T or C (from N-addition or from the 5′ end of the IGHJ gene) will result in a functional sequence, for TAA and TAG are stop codons. Addition of C returns the sequence to its original state, while addition of T results in an alternative tyrosine codon. In this and other cases, the nucleotide sequences of the gene ends limit the diversity that results from exonuclease removals.

## B Cell Lineages and T Cell Clonotypes in the Antigen-Specific Response

Biases that we have described in immunoglobulin V, D, and J gene usage mean that at least seven orders of magnitude separate the probabilities that the most likely and the least likely combinations of recombining genes will be generated in the bone marrow. Many additional orders of magnitude separate the most likely from the least likely heavy and light chain pairs. The least likely BCRs are so unlikely to be generated in the bone marrow that they almost certainly will never be seen in an individual’s lifetime. The most likely BCRs, on the other hand, may be so readily generated that they are always present within the repertoire at high copy number. These high copy-number sequences are likely to utilize a relative handful of the available germline genes, and to have been subject to minimal processing. TdT adds, on average, around 6 nucleotides between joining genes, and 30 or more nucleotides may occasionally be added to the VD, DJ, and VJ junctions, but it is highly likely that no more than two nucleotides will be added. Even long heavy chain CDR3 are likely to be the result of long germline sequences rather than the result of long N-REGIONS (47). Six or more nucleotides may be removed from the 3′ end of the IGHV gene, but most sequences lose no more than two or three IGHV nucleotides, and many sequences lose no nucleotides at all.

Without the added diversity that comes from D genes, the kappa and lambda repertoires are strongly shaped by biased gene usage and minimal processing and the diversity of the repertoires is surprisingly limited. The light chain repertoires are dominated by a very small number of amino acid sequences, and this dominance is so extreme that even in the days of Sanger sequencing, identical light chain gene rearrangements were reported by separate studies from independent laboratories (20). The theoretical diversity of the kappa repertoire has been estimated to be as high as 4 × 10^24^ unique nucleotide sequences (91). However analysis of kappa sequences generated from single individuals by high-throughput sequencing suggest the repertoire may include less than 10^4^ unique amino acid sequences (55), and some of these sequences may be seen in over 1% of all kappa-bearing BCR (55). The diversity of the expressed lambda repertoire has recently been shown to be similarly restricted (63).

Although the heavy chain repertoire has much greater diversity then the light chain repertoire, repertoire shaping may be sufficiently extreme that some heavy chain sequences, and even some BCR will be present at high copy number in the repertoire of every individual. We are not aware of identical heavy chain sequences being amplified from multiple individuals, but highly similar “stereotypical” sequences have been found amongst leukemic clones of individuals with chronic lymphocytic leukemia (92). These stereotypical sequences differ through the stochastic processes of somatic hypermutation, but they appear to have evolved from cells expressing highly similar BCR within the naïve B cell repertoires of different individuals. Antigen selection, which may be associated with the pathogenesis of this condition (93), could be selecting and therefore revealing high copy-number heavy chain sequences.

The antigen specificity of most heavy and light chain sequences remain unclear, for it is only very recently that antigen-specific human B cells have been isolated and their BCRs investigated. The isolation of antigen-specific plasmablasts from the peripheral blood shortly after vaccination was first used to produce monoclonal human antibodies (94). These cells express BCR genes that are at once similar, as a consequence of their shared origins, yet highly divergent, as a result of the process of somatic point mutation. Together they make up a B cell clone lineage. High-throughput sequencing has since been used to identify clone lineages after booster shots with the influenza vaccine (95) and the pneumococcal vaccine (96). B cell lineages producing broadly neutralizing antibodies to HIV have also been identified using high-throughput sequencing (97). However this handful of studies of antigen-specific B cells in humans has not identified lineages that are shared between individuals. Highly similar BCR heavy chain sequences have recently been identified using high-throughput sequencing of PBMC from multiple individuals with acute symptomatic dengue (98). Although the specificities of these sequences were not determined, such lineages were not identified in uninfected individuals. These may therefore be the first antigen-specific heavy chain “public lineages” to be identified. The extent to which the response to specific antigen more generally involves such “public lineages” remains to be determined.

In contrast to the paucity of studies of antigen-specific B cells, antigen-specific TCRs have been investigated in the human repertoire for over 20 years. Early studies revealed that the immune response to specific antigen, in HLA-matched individuals, can include sets of T cells sharing identical or highly similar TCR α- and β-chains (99–101). The development of techniques for the creation of MHC peptide tetramer complexes has facilitated the identification of antigen-specific T cells by flow cytometry (102). This has allowed the detailed investigation of dominant sequence sets and these studies gave rise to the notions of public and private T cell “clonotypes.” Public clonotypes are defined as VDJ amino acid sequences that are dominant and identical, or nearly identical, in multiple individuals. Private clonotypes, in contrast, are idiosyncratic. The apparently antigen-driven emergence of public B cell lineages in chronic lymphocytic B cell leukemia also has parallels amongst T cell leukemias. Studies of T cell large granular lymphocyte leukemias have identified a public clonotype in individuals with the shared DRB1*0701 HLA type (103). This same clonotype was independently identified in DRB1*0701^+^ individuals who were infected with human cytomegalovirus (104), suggesting that antigen-driven pathogenesis may be expanding and revealing this public clonotype.

To understand the reasons for the emergence of particular clonotypes, the naïve repertoire must be better understood. Enrichment techniques have recently been developed which when combined with MHC peptide tetramer technology allows extremely rare peptide-specific naïve murine T cells to be identified (105). Using this approach in humans, naïve CD8^+^ T cells specific for peptide-MHC have been shown to range from 0.6 to 500 cells per million cells (106, 107) and CD4+ T cells to range from 0.2 to 10 per million cells (107). Most of the cells within identified sets of antigen-specific murine T cells express unique TCRs (105), but clonal diversity within identified human cell populations remains unclear. It is likely though that in the much larger human T cell compartment, many circulating T cells could carry identical TCRs. This should ensure that early adaptive responses to these antigens are robust, for the strength of the response to antigen has been shown to reflect the size of the antigen-specific naïve T cell population (105).

The presence of particular public TCR clonotypes have not yet been reported within the naïve human TCR repertoire. Discussion of the emergence of such TCR clonotypes in an antigen-specific response has therefore been driven principally by analyses of their nucleotide and amino acid features, and the phenomenon of convergent recombination has been invoked to explain public clonotypes (108, 109). Many public TCR clonotypes are divergent at the nucleotide level, but identical at the amino acid level. This results from the fact that particular amino acid sequences can arise from multiple, variant nucleotide sequences, and that these nucleotide sequences in turn can sometimes be formed by different genes with varying levels of gene processing and nucleotide addition. Such convergent recombination will certainly contribute to the presence of multiple copies of particular amino acid clonotypes within an individual’s repertoire, but arguably, it is unlikely to increase the likelihood of one clonotype over another by more than one or two orders of magnitude.

More recently the role of biases in gene usage and in the recombination process have been identified as an alternative source of public clonotypes (110). The biases in the usage of TCR V, D, and J genes are less pronounced than is the case for the BCR genes. This is the result of the lack of substantial germline diversity within the sets of TRBD and TRDD genes, and because the TRBJ and TRDJ genes lack the strong usage biases that are seen amongst the IGHJ genes. Nevertheless biases in the usage of TCR genes are still likely to ensure that the probabilities of the generation of the least likely and the most likely V(D)J combination seen in αβ and γδ TCR differ by many orders of magnitude. It has also been pointed out that many public clonotypes have short CDR3 loops that are mainly encoded by germline-derived nucleotides rather then TdT-derived nucleotides (110). The contribution this may make to the formation of T cell clonotypes is harder to judge, because of the lack of detailed analysis of these processes, in the context of the TCR repertoire. However lessons from analysis of the BCR repertoire give strong credence to this hypothesis.

Both the BCR and the TCR repertoires have been the subject of considerable study and even greater speculation over many decades. High-throughput sequencing is now revealing their separate secrets at a gratifying rate. Our understanding of the shaping of the BCR and TCR repertoires will now surely move faster if a greater dialog commences between researchers on the two sides of the lymphocyte divide. BCR repertoire studies will be transformed when greater attention is paid to antigen-specific lineages. TCR repertoire studies, in turn, could benefit from the lessons of the BCR repertoire, which suggest that the analysis of full-length V(D)J rearrangements, and detailed analysis of the nucleotide elements within the CDR3, can help explain the shaping of the repertoire.

## Conflict of Interest Statement

The authors declare that the research was conducted in the absence of any commercial or financial relationships that could be construed as a potential conflict of interest.
